# Vaccination with *Toxoplasma* lysate antigen or its encapsulated niosomes form immunomodulates adjuvant-induced arthritis through JAK3 downregulation

**DOI:** 10.1007/s10787-023-01267-0

**Published:** 2023-06-30

**Authors:** Sally S. Hassouna, Eman A. Allam, Eman Sheta, Gehan A. M. Khodear, Marwa I. Khedr, Safaa I. Khedr, Maha M. Gomaa

**Affiliations:** 1 Internal Medicine Department, Rheumatology and Immunology Unit, Faculty of Medicine, Alexandria University, Alexandria, Egypt; 2 Medical Physiology Department, Faculty of Medicine, Alexandria University, Alexandria, Egypt; 3 Pathology Department, Faculty of Medicine, Alexandria University, Alexandria, Egypt; 4 Medical Technology Center, Medical Research Institute, Alexandria University, Alexandria, Egypt; 5 Medical Biochemistry Department, Faculty of Medicine, Alexandria University, Alexandria, Egypt; 6 Medical Parasitology Department, Faculty of Medicine, Alexandria University, Alexandria, Egypt

**Keywords:** Arthritis, *Toxoplasma* lysate antigen, *Toxoplasma* lysate antigen-encapsulated niosomes, Niosomes, Prednisolone and Janus kinase 3

## Abstract

**Background:**

Inflammatory autoimmune arthritis like that present in rheumatoid arthritis (RA) is treated by medications with many side effects. This study was a trial to benefit from *Toxoplasma* immune-modulatory effects on its host to treat arthritis in rat model resembling joints affection of RA. To avoid hazards of infection, *Toxoplasma* lysate antigen (TLA) was given instead of the whole infection, in addition to giving its encapsulated niosomes form, assuming that it would enhance the effect of TLA alone, to compare effects of both on disease activity with that of prednisolone.

**Methods:**

Swiss albino rats were divided into 6 groups: normal control group and the remaining 5 groups were injected by CFA adjuvant to induce arthritis; one of those groups was the untreated model. Each of the other groups received one of the following (TLA, TLA-encapsulated niosomes, prednisolone or niosomes) for comparison of their results. Inflammatory markers measured at the end of the experiment were: interleukin 17 (IL-17), IL-10 and CRP by ELISA technique; histopathological assessment of the biopsied hind paw joints was done and also, Janus kinase 3 (JAK3) expression was assessed by immunohistochemistry.

**Results:**

TLA and TLA-encapsulated niosomes both mitigated the signs of clinical and histopathological arthritis and were having anti-inflammatory effects (decreased CRP, IL-17 and JAK3 expressions, while increased IL-10 levels) with better effects in TLA-encapsulated niosomes-treated RA group, both groups’ results were comparable to prednisolone. Niosomes also gave some anti-inflammatory effects but were mild in comparison to TLA and TLA-encapsulated niosomes.

**Conclusion:**

Vaccination with both TLA and TLA-encapsulated niosomes for the first time in adjuvant-induced arthritis ameliorated the disease through diversion of immune system and JAK3 downregulation. Both vaccinations should be further tested to evaluate the possibility of their introduction for disease treatment and in other autoimmune diseases.

**Supplementary Information:**

The online version contains supplementary material available at 10.1007/s10787-023-01267-0.

## Introduction

Inflammatory autoimmune arthritis is most commonly present in rheumatoid arthritis (RA), which is an autoimmune disorder that results in warm, painful and swollen joints. These manifestations and joint stiffness worsen after rest. Mostly, the wrists and hands joints are involved, typically on both body sides. Extra-articular manifestations may be present including anemia, inflammation around lungs, and heart. Fever and fatigue may also be present. Symptoms come gradually over weeks or months. Pathogenesis of RA is still not clear, but it is believed to be due to genetic and environmental factors involving the immune system which attacks the joints (Institute and of Arthritis and Musculoskeletal and Skin Diseases (NIH) 2014; Majithia and Geraci 2007).

Jannus kinase 3 (JAK3) are cytosolic tyrosine kinases specifically allied to cytokine receptors. Because cytokine receptor proteins do not have enzymatic activity, they are dependent on JAKs to begin signaling after binding of their ligands. JAK3 appeared to play crucial roles in immune and nonimmune physiology since tofacitinib, a selective JAK3 inhibitor is a treatment for certain inflammatory diseases and has an immunosuppressive activity in RA (Kumar et al. 2021).

Interleukin 17 (IL-17) contributes in early and late induction stages of several chronic inflammatory diseases including RA where IL-17 A triggers synovial changes leading to synovitis and causes local inflammation (Miossec and Kolls 2012; Aggarwal and Gurney 2002). On the other hand, interleukin 10 (IL-10), known as human cytokine synthesis inhibitory factor, is an anti-inflammatory cytokine which is increased in remission of inflammatory diseases (Eskdale et al. 1997).

RA treatment aims to reduce pain and inflammation, and to improve the patient’s functioning (Institute and for Health and Care Excellence (NICE) 2020). Steroids, NSAIDs and pain medications are frequently used to alleviate symptoms. (Institute and of Arthritis and Musculoskeletal and Skin Diseases (NIH), 2014) While, disease-modifying anti-rheumatic drugs (DMARDs) such as methotrexate and hydroxychloroquine may slow the disease progression (Institute and of Arthritis and Musculoskeletal and Skin Diseases (NIH), 2014). Biological DMARDs also may be used when other treatments are not so effective alone (Singh et al. 2015).

Serious side effects of RA medications make it necessary to search for an aiding treatment with lesser side effects (Singh et al. 2011; Stuart 2020). A treatment that depends on the natural diversion of immune system such as introducing a foreign body, i.e., a parasite (protozoan) or an antigen of this parasite, is called “immunomodulation” (Maizels 2009).

Immunomodulation means using therapeutic interventions aiming to change the immune response either enhancement of immune response which is desirable in immunodeficiency to prevent infections, to treat established infections or cancer, or to weaken the immune response as in autoimmunity, organ transplantation and allergies (Lüder et al. 2010; Hasseldam et al. 2013; Engwerda et al. 2014).

Parasitic worms, as a whole or their antigens, appeared to modulate some autoimmune diseases including RA (Jankovic et al. 2007); this is due to secretion of immunomodulatory products that change the host immune response to be able to survive in their hosts for long time (Lüder et al. 2010) through Toll-like receptor pathways modulation and regulatory immune responses induction with Th2 pro-inflammatory responses “modified Th2 response” (Wilson et al. 2005; Smallwood et al. 2017; Jackson et al. 2009).

Protozoa to which *Toxoplasma* belongs also undergo several mechanisms to escape host’s immunity, that leads to decreased pathological insults caused by the parasite to the host tissue. *Toxoplasma gondii* (*T. gondii)* produces virulence factors into the host cells that inhibit acquired and innate immune responses of the host (Lima and Lodoen 2019). Rhoptry kinase 16 (ROP16) a major virulence factor molecule produced by the parasite, can induce macrophages activation at an early infection phase through IL-4 and IL-10 expression stimulation, leading to a Th2- response promotion (Jensen et al. 2013). Moreover, *Toxoplasma* infection showed alleviating effect on experimental model of arthritis (Washino et al. 2012; Hafez et al. 2020).

Niosomes are vesicles based on non-ionic surfactant mostly with cholesterol incorporation. Niosomes have good penetrating ability and can carry both hydrophilic and lipophilic drugs and act as a novel drug delivery system to improve drug delivery and to support sustained effect (Moghassemi and Hadjizadeh 2014; Mujeeb et al. 2015).

Side effects of autoimmune arthritis medications and *Toxoplasma* infection effects on improving the disease give rise to the following questions. Would *Toxoplasma* lysate antigen (TLA), which is a crude T. gondii antigen that was used as a vaccine in previous experiments to protect against toxoplasmosis as it covers a wide range of protective parasitic antigens (multi-antigenic) (EL-Malky 2014), have a therapeutic effect on adjuvant-induced arthritis? Would niosomes-encapsulated TLA enhance this effect if present? Would one or both of them substitute for or be adjuvant/adjuvants for immune-suppressant agents that are already known for disease treatment?

## Materials and methods

### Drugs and chemicals

Complete Freund adjuvant (CFA) which consists of heat-killed *Mycobacterium butyricum* in mineral oil was purchased from Sigma-Aldrich, Egypt (Cat. No: F5881).

Prednisolone in the form of prednisolone sodium phosphate was purchased from Borg Pharmaceutical industries, Egypt.

### Parasite and maintenance

Toxoplasma gondii RH HXGPRT (-) virulent strain was obtained from the laboratory of Medical Parasitology Department, Alexandria Faculty of Medicine, Egypt. It was maintained by serial inoculation of tachyzoites in albino mice every five days through intraperitoneal route. One milliliter of sterile isotonic saline (0.9%) was injected into the peritoneal cavity of the infected mice five days post-infection (PI). The peritoneal fluid was withdrawn and examined for tachyzoites under light microscope (× 400). This process was repeated three times and the obtained tachyzoites were collected for antigen preparation (Temsahy et al. 2016).

### Preparation of *Toxoplasma* lysate antigen

The collected tachyzoites were sedimented through centrifugation (2,000 rpm for 10 min), then washed in phosphate buffer saline (PBS, PH 7.4) three times and undergone eight cycles each of 10 s of sonication with 5 s in between for cooling at 4 °C. *Toxoplasma* lysate was centrifuged (1,000 rpm for 20 min).The supernatant material which is the TLA was collected, protein concentration was determined by a commercial total protein determination kit and then TLA was put at − 20 °C for preservation (Temsahy et al. 2016).

### Preparation of niosomes and TLA-encapsulated niosomes

Preparation of TLA suspension was done in PBS (7 Ml) (Pardakhty et al. 2012). Niosomes were prepared by thin film hydration method. Cholesterol and Span 60 in molar ratio of 6:7 was dissolved in chloroform and ethanol mixture (10 Ml). Evaporation at 55 °C under reduced pressure (200 mmHg) in a rotator evaporator; for 15 min; was done to extract the solvent and to leave the nano-droplets. A thin film was obtained (formation of nanocarriers by macromolecules precipitation). This dry lipid film was then hydrated with PBS (10 Ml, Ph 7.4), then underwent shaking in the evaporator at 55 °C at a low speed for 15 min and hand shaking at room temperature for another 15 min to have a lipid suspension. Sonication of the particles to be down sized was then done.

The same method of niosomes preparation was followed, but TLA was added to span 60 and cholesterol in the first step, to prepare TLA-encapsulated niosomes. Then, its lyophilization (by freezing 50 Ml of the TLA-encapsulated niosomes suspension in liquid nitrogen and drying it by the vacuum freeze-drying machine at 26.5 Pa pressure) were performed. After that TLA-encapsulated niosomes and niosomes characterization was done through Beckman coulter laser light scattering for analysis of particle size. Colloidal dispersion stability was determined by Zeta Potential Determination, Physical size and shape of niosomes and TLA- encapsulated niosomes were determined using TEM. Ratio between the TLA concentration in the niosomes and its concentration added to the system was calculated to express the niosomes’ encapsulation efficiency (EE) when the TLA was carried on the produced niosomes (G D.B. and P V.L. 2020). Zeta potential, the colloidal dispersion stability indicator, was determined using laser doppler anemometry and a zetasizer. Niosomes and TLA-encapsulated niosomes were diluted in phosphate buffer of Ph 6.5. Electrophoretic velocity of the studied particles was observed by 150 Millivolts (Mv) electric field. All procedures were made at 25ºC at the same ionic concentration in triplicate. Particle’s suspension was diluted with distilled water ten times. It was deposited onto a 400-mesh copper grid coated with carbon in a drop-wise, then was allowed to dry in air before examination under microscope (G D.B., P V.L. 2020). Fourier transforms infrared (FTIR) spectroscopy was used to measure the IR radiation absorption by materials as atoms vibrate at their bonds; it is used to identify types of bonds, structures and functional groups of organic and inorganic compounds obtained using a Shimadzu FTIR-8400S (Tokyo, Japan).

### Experimental animals

Approval of institutional Ethics Committee of Faculty of Medicine, Alexandria University, Egypt for this experiment was obtained (IRB code: 00012098, FWA: No.: 00018699; International Council of Laboratory Animal science organization (ICLAS) membership with serial number of registration (0305205)).

Forty-two albino male rats purchased from Medical Physiology Department, animal research laboratory, Faculty of Medicine, Alexandria University, Egypt were housed in plastic cages (each cage contained 4 rats) at 23 ± 3 °C in a stable humidity and a natural day and night cycle at the research laboratory while having a free access to tap water and their standard food. Rats’ weights ranged between 120 and 140 g at the beginning of the experiment.

### Study design

#### Induction of arthritis model and application of treatments

All study animals were left for one week for the purpose of acclimatization to housing. Then, arthritis induction resembling that in RA was done in 35 rats by 0.1 ml CFA subcutaneous injection into right hind paw plantar surface. Booster doses of 0.1 ml intra-dermal injection were given into the tail root on the same and the following days (Darwish et al. 2013; Shirani et al. 2021).

The remaining 7 rats were constituting the normal control group (group A) and received mineral oil subcutaneous injection of the same volume as CFA at the same sites with no inflammation-inducing agent. All injections were done after sterilization of skin using betadine antiseptic solution.

Paw injection was done on day 0 of the experiment and then, local arthritis signs appeared on day 1 in the CFA-injected paws; those rats with arthritis were then divided into five groups (7 rats in each group): group B: a group of arthritis which received no treatment but oral gum acacia solution as placebo treatment given on a daily basis; group C: a group of rats which received TLA alone (Mujeeb et al. 2015); group D: a group of rats with arthritis injected which received TLA-encapsulated niosomes; group E: a group of rats which received arthritis injected with niosomes only; and group F: a group of arthritis treated which received a low dose of prednisolone syrup (1.25 mg/kg/day) (Kuncha et al. 2014). Oral treatments were given from day 1 and continued till the study end at day 28 using a gastric tube (gavage). Pain or discomfort was minimized by taking adequate measures. TLA, TLA-encapsulated niosomes and niosomes were injected subcutaneously, each was given to its targeted group with a dose of 120 μg protein/rat for TLA and TLA-encapsulated niosomes and 100 μl for niosomes. This was performed twice; on day one and day 14 of the experiment. (EL-Malky 2014).

#### Clinical assessment and scoring of arthritis progression

Clinical assessment, paw thickness measuring (using a Vernier caliper) for objective confirmation of foot edema and inflammation (at nearly 0.02 mm) (Osada et al. 2009) and arthritis scoring were performed on days 0, 7, 14, 21 and 28. Paws were examined for erythema and swelling; then arthritis score was given by the mean of the recorded scores for each paw with a 5-point scale from 0 as a minimum score to 4 as a maximum score for each paw. Maximum arthritis score was 12 for the three non-injected paws where secondary arthritis occurs. Arthritis was indicated on a score greater than 6 (Cremer et al. 1990).

#### Weight measurement

The weight (in grams) was plotted on a chart on days 0, 7, 14, 21, 28; this was done for the assessment of arthritis severity, treatments’ effectiveness, and also to calculate the dose of prednisolone.

#### Study termination

On day 29, the study was terminated. Rats were anesthetized with ether inhalation after fasting overnight, then blood samples were picked up through cardiac puncture, afterwards the animals were killed. Serum separation was done for the obtained blood samples after being put in non-heparinized test tubes; then centrifugation for 15 min at 3000 rpm was done. Storage of the serum samples at − 20 °C was done for biochemical analysis later. The right hind paws were put in 10% formalin for histopathological examination.

### Measurement of serum levels of interleukin 10 (IL-10), IL-17 and CRP

ELISA was conducted to measure the levels of serum IL-10 (Cat No. In-Ra0655, Innova Biotech Co., Ltd, China), IL-17 (Cat No. In-Ra0662, Innova Biotech Co., Ltd, China) and CRP (Cat No. In-Ra0574, Innova Biotech Co., Ltd, China) for rats in all groups. The analysis was done in accordance with the manufacturer’s instructions.

#### Histopathological examination procedures

Biopsied right hind paws were cut along its longitudinal axis and embedded on plastic cassettes. All collected paws were processed in different degrees of alcohol, xylene then embedded in paraffin. A microtome (leica, HistoCore BIOCUT—Manual Rotary Microtome, Germany) was used to cut 5-micron sections. They were mounted on glass slides and stained by hematoxylin and eosin. Slides were then examined by light microscopy (Olympus, CX23, Tokyo, Japan) for any pathologic changes. Severity of joint injury was assessed according to four histologic parameters. First, the joint and/or bone erosions which were seen as defects in the articular cartilage or necrosis of underlying bone. Second, synovial infiltration by inflammatory cells. Third, pannus formation which is formed of severe inflammation within fibrotic tissue. Finally, synovial hyperplasia, which is detected as proliferation of synovial layer lining the joint space. Each parameter was examined alone and graded from 0 to 4. At the end, the sum of the four parameters’ score was calculated out of 16(Silva et al. 2004).

#### Immunohistochemistry

Paraffin blocks of hind paw were then cut on positively charged slides. They were stained using DAB technique by anti-JAK3 primary antibody (#MBS2561404, polyclonal rabbit antibody, my BioSource, San Diego, USA). The concentration used was 1:75 by DAKO autostainer (DAKO, Link 48). Citrate buffer was used as a retrieval for 20 min. Stained slides were then assessed for cytoplasmic staining in synovial and inflammatory cells according to walker et al. (Walker et al. 2006) The staining was scored in 4 grades: grade 0, 1, 2, 3 and 4 if less than 5%, 5–25%, 26–50%, 51–75% or more than 75% of cells are positive, respectively. Scoring was done in all paw joints examined. Then, a mean final score was given.

### Statistical analysis of the experiment findings

Data were analyzed by means of IBM SPSS software package (Armonk, NY: IBM Corp) of version 20.0. Shapiro–Wilk test was used to test for continuous data normality. For normally distributed quantitative data, they were used in ranges of minimum and maximum, median, mean and standard deviation. One-way ANOVA test followed by Tukey Post Hoc test for pairwise comparison was used for comparing the studied groups. The Kruskal–Wallis test was applied to compare groups for quantitative variables that are not normally distributed, followed by Dunn’s test for multiple comparisons test (Post Hoc test) to provide a pairwise comparison. Significance of results was judged at the level of 5%.

## Results

### Determination of entrapment efficiency (EE) of niosomes

The encapsulation efficiency of the crude *Toxoplasma* lysate vaccine (CTLV) encapsulated with niosomes was determined by analyzing the supernatant of the final emulsion once the CTLV encapsulated with niosomes were removed from it by centrifugation at 16,000 rpm for 30 min. For the estimation of CTLV present in the supernatant, the absorbance was measured spectrophotometrically at 315 nm using UV–Vis spectrophotometer (UNICAM UV–Vis spectrometry model UV5-220) and the amount of CTLV present was calculated from calibration curves of concentration versus absorbance with known standards of the CTLV. The amount of the CTLV encapsulated and the percentage of encapsulation in the nanocarriers is given by: Encapsulated CTLV concentration = (Total amount of CTLV –(Free amount of CTLV), encapsulation% = (Total amount of CTLV-Free amount of TLV) × 100 Total amount of CTLV, Encapsulation% = (80 ug-20 ug) × 100 80 ug = 75%

### Characterization of niosomes and TLA-encapsulated niosomes is shown in (Fig. 1)

**Fig. 1:**
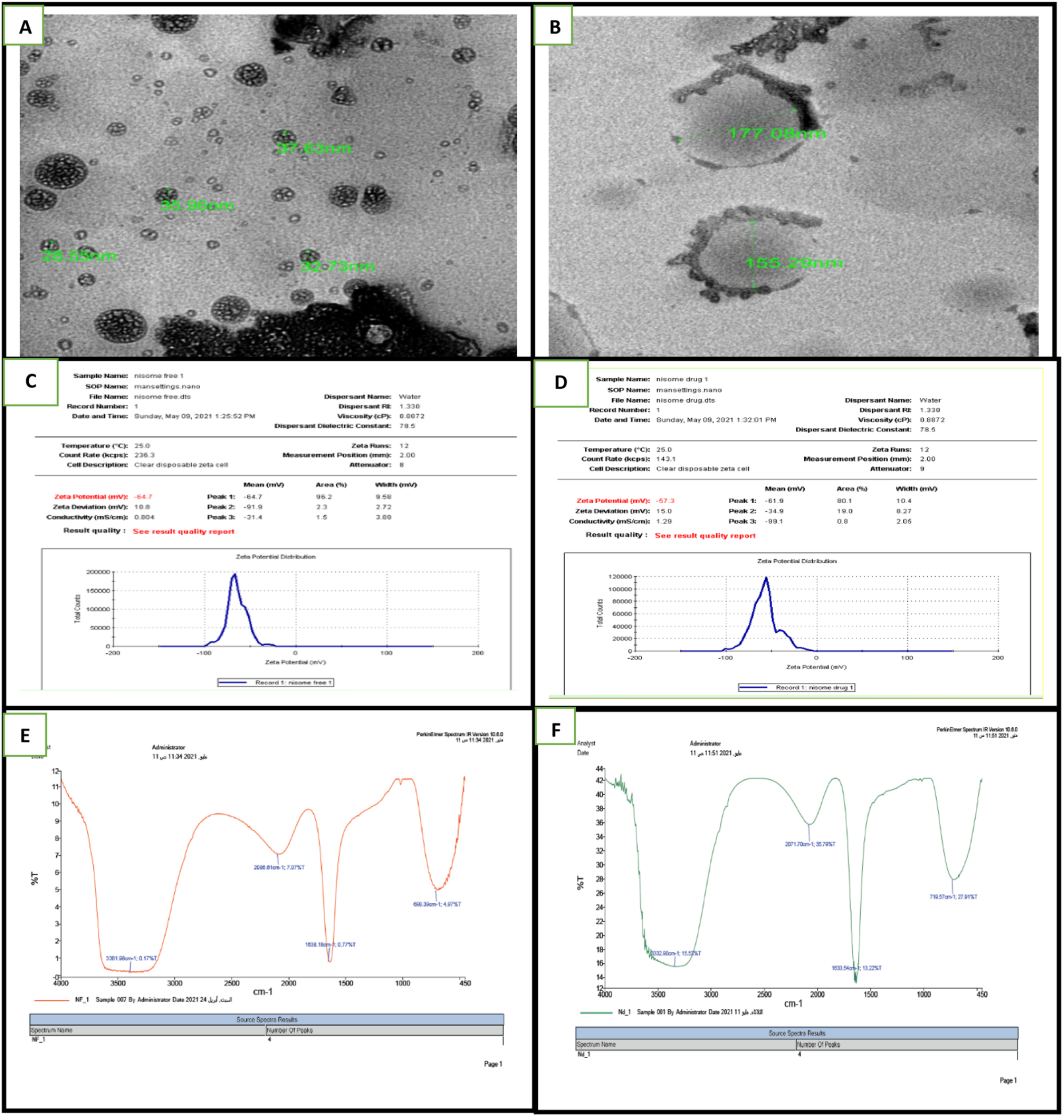
A The Transmission Electron Micrograph (TEM) of the free niosomes and B TLA-encapsulated niosomes were spherical or oval in shape. Average sizes of niosomes and TLA-encapsulated niosomes were 33.71, 164.12 nm. Niosomes were negatively charged with zeta potentials were − 64.7 and − 57.3 mV for C Niosomes and D TLA-encapsulated niosomes. FTIR of E Free niosomes and F TLA-encapsulated niosomes

#### Assessment of arthritis induction and progression

On day 1, inflammatory signs (paw redness, hotness, swelling and limitation of paw movement expressed by foot dragging) appeared in the paw injected with the adjuvant and remained with variability till the experiment end. The non-injected paws had the same inflammatory signs but this started on day 14 till the experiment end.

**Fig. 2:**
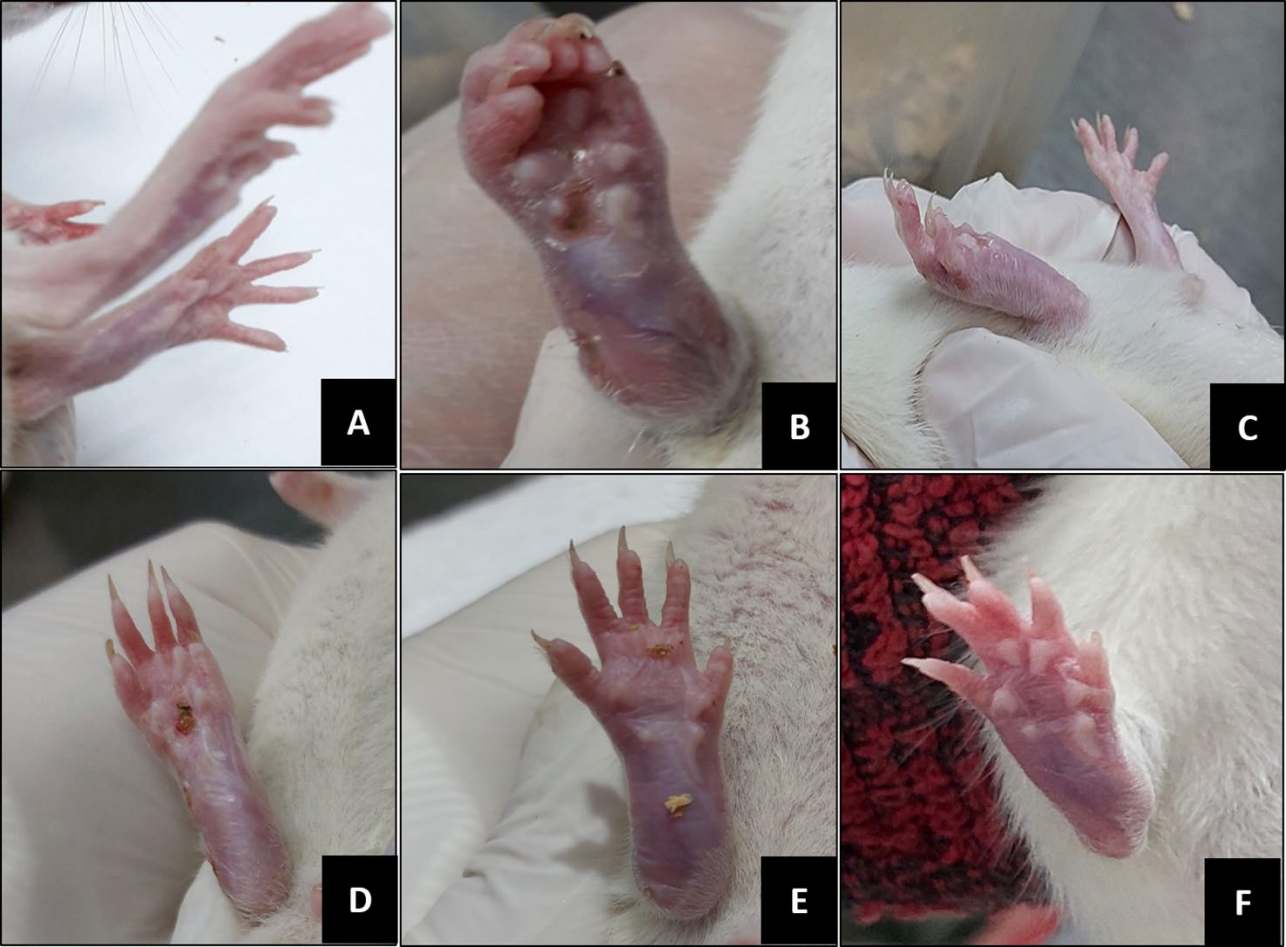
Clinical appearance of right hind paw joints of different groups. **A** Normal control group, **B** Untreated arthritis group, **C** TLA-treated arthritis group, **D** TLA-encapsulated niosomes-treated arthritis group, **E** Niosomes treated arthritis group

The inflammatory signs course was progressive in the untreated group. While, groups which received vaccination with TLA and TLA-encapsulated niosomes besides the group treated with prednisolone showed improvement of inflammatory signs. Rats with arthritis which received niosomes showed only mild improvement of arthritis signs. (Fig. 2).

##### Arthritis scoring

**Fig. 3:**
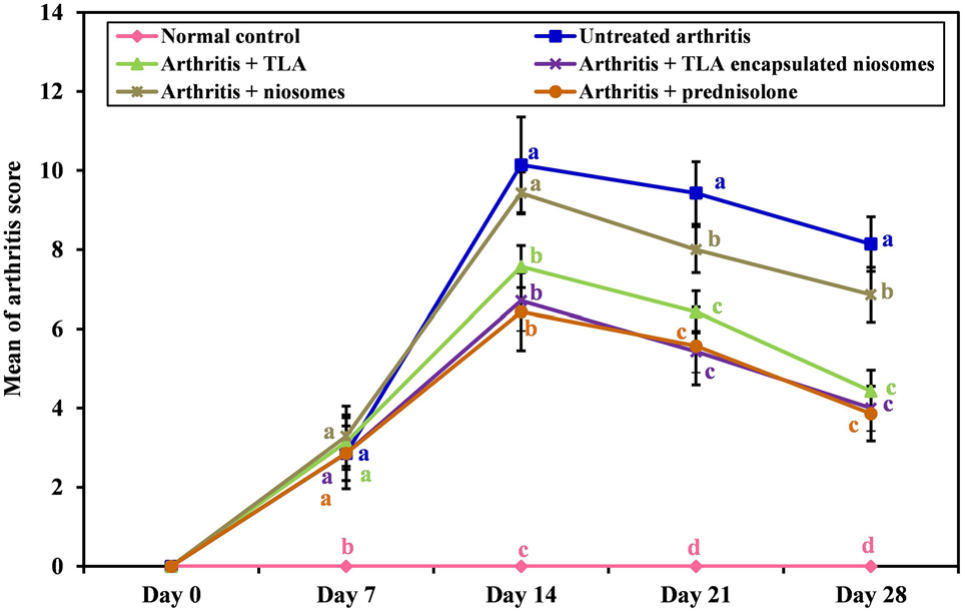
Arthritis score of different groups. Means with any common letter from “a” to “d” are not significantly different, i.e., means with totally different letters “a” to “d” are significantly different

On days 14, 21 and 28, arthritis score for secondary arthritis in non-injected paws wa significantly different than the normal control group from its increased value in all other groups *p* =  < 0.001 till the end of experiment and between untreated group arthritis score and other groups which were receiving treatments (*p* =  < 0.001) except for the group of arthritis which was receiving niosomes only; it was not significant on day 14 but was only significant on day 21 (*p* = 0.002) and on day 28 (*p* = 0.003); also, the group which received niosomes was significantly different in arthritis score from other treatments (*p* value = 0.001 on day 14 and *p* =  < 0.001on day 21 in TLA group and < 0.001 on days 14, 21 and 28 with TLA-encapsulated niosomes and prednisolone), while no significant difference in arthritis score among other treatments (neither TLA with TLA-encapsulated niosomes nor either of both treatments with prednisolone). (Fig. 3).

##### Body weight measurement

**Fig. 4:**
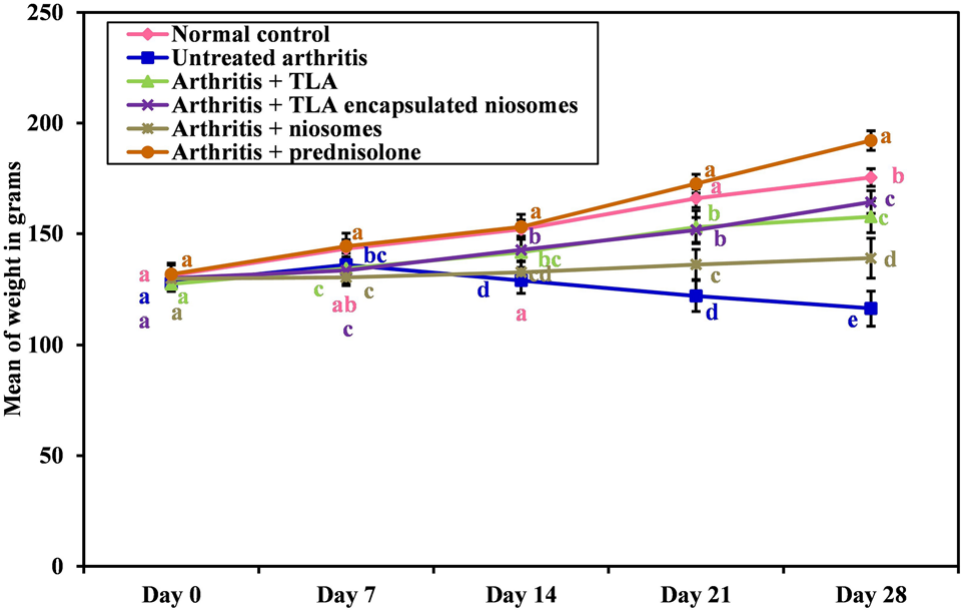
Weight of rats in the studied groups. Means with any common letter from “a” to “d” are not significantly different, i.e., means with totally different letters “a” to “d” are significantly different

Rats in all groups were at the same range of weight at the beginning of the experiment without any significant difference. At the end of the experiment weight in normal control group was significantly higher than rats’ weights in other groups (*p* =  < 0.001 with untreated arthritis group (whose weight was significantly lower than all other groups *p* =  < 0.001), *p* =  < 0.001 for TLA and TLA-encapsulated niosomes-treated arthritis groups and *p* = 0.031 for niosomes-treated arthritis group) except for the group which received prednisolone where weight increased than that in normal group (*p* =  < 0.001). Weight in prednisolone-treated group was significantly higher than those in other treatments *p* =  < 0.001. While weight in niosomes-treated arthritis group was decreased that those in other treatment groups *p* =  < 0.001. TLA and TLA-encapsulated niosomes-treated arthritis groups did not have significant difference in weight. (Fig. 4).

### Changes in the inflammatory markers (IL-10, IL-17 and CRP)

The anti-inflammatory cytokine IL-10 was significantly decreased (53% decrease, *P* =  < 0.001), while the inflammatory markers IL-17 (2.8-fold increase, *P* =  < 0.001) and CRP (9.38 folds increase, *P* =  < 0.001) increased in the untreated arthritis group versus the control group. All types of treatment TLA, TLA-encapsulated niosomes, niosomes and prednisolone were effective to increase IL-10 (37% increase, *P* = 0.001 in TLA group), (45% increase, *P* =  < 0.001 in TLA-encapsulated niosomes group), (21% increase, *P* =  < 0.047 in niosomes group) and (42% increase, *P* =  < 0.001 in prednisolone group), versus untreated rats. For IL-17, it significantly decreased (41% decrease, *P* = 0.001 in TLA group), (52% decrease, *P* =  < 0.001 in TLA-encapsulated niosomes group), (25% decrease, *P* =  < 0.001 in niosomes group) and (38% decrease, *P* =  < 0.001 in prednisolone group) versus untreated rats. The same was applied on CRP, as all treatments significantly decreased its level (71% decrease, *P* = 0.001 in TLA group), (87% decrease, *P* =  < 0.001 in TLA-encapsulated niosomes group), (48% decrease, *P* =  < 0.001 in niosomes group) and (80% decrease, *P* =  < 0.001 in prednisolone group) versus untreated rats. Regarding the comparison between treatment groups, niosomes-treated group exhibited a significantly higher level of IL-17 than all other treated groups (*P* = 0.003 for TLA, *P* =  < 0.001 for TLA-encapsulated niosomes, *P* = 0.023 for prednisolone). CRP levels were significantly higher in niosomes-treated group than TLA-encapsulated niosomes (*P* =  < 0.001) and prednisolone (*P* = 0.004)-treated groups. As regards IL-10 levels comparison between treatment groups, niosomes-treated group exhibited a significantly lower level than all other treated groups (*P* = 0.008 for TLA, *P* =  < 0.001 for TLA-encapsulated niosomes, *P* =  < 0.001 for prednisolone). (Supplementary Fig. 2 and 3).

#### Histopathological examination

In all hind paws examined, multiple joints were assessed per section. No pathologic changes were detected in the normal group. All small joints were covered by a thick intact smooth cartilaginous cap with viable underlying bone. Synovium is seen covered by one layer of flat synovial cells without remarkable inflammatory cells. whereas the untreated arthritis group showed different pathologic changes. The synovium was thickened, hyperplastic and papillary in some areas. All were covered by proliferating 2–4 layers of synovial cells. Sub-synovial tissues showed severe inflammation which was composed mainly of lymphocytes, plasma cells admixed with macrophages and giant cells. pannus formation was seen as wide areas of fibrosis and it focally invaded the bony areas. The articular cartilage was thinned out with focal total loss. Chondrocytes were degenerated with multiple empty lacunae. The pathologic arthritis total score in untreated arthritis group ranged between 12 and 14 and it was statistically significant in comparison to normal group (*p* =  < 0.001).

Niosomes-treated arthritis group showed only minimal improvement of joint injury in comparison to untreated arthritis group. It was seen in the decrease of pannus formation and cartilage erosions. However, the inflammation and synovial hyperplasia were still detected. The total arthritis score in niosomes-treated arthritis ranged between 9 and 12 and this was statistically insignificant in comparison to untreated arthritis (*p* = 0.349).

Arthritis group which was treated by TLA showed moderate improvement of arthritis. The inflammation decreased to be mild to moderate. Synovial infiltration by lymphocytes and plasma cells was still detected. No pannus was seen in most of the examined joints. The cartilage irregularities and erosions were seen only focally and no bone loss was detected. Synovium was still hyperplastic in some areas. The total arthritis score in this group ranged between 1 and 6 and this improvement was statistically significant in comparison to arthritis model (*P* = 0.003) and niosomes-treated arthritis (*P* = 0.042).

Adding the TLA to niosomes improved its anti-inflammatory effect. No pannus formation was seen in any of examined paws. The inflammation and synovial hyperplasia were only minimal. No cartilaginous loss was detected. The total arthritis score ranged between 1 and 6. This effect was statistically significant in comparison to untreated arthritis and niosomes treated arthritis (*P* = 0.003, 0.042, respectively). However, no statistically significant effect was seen in comparison between TLA and TLA-encapsulated niosomes treated groups (*p* = 1.000).

**Fig. 5:**
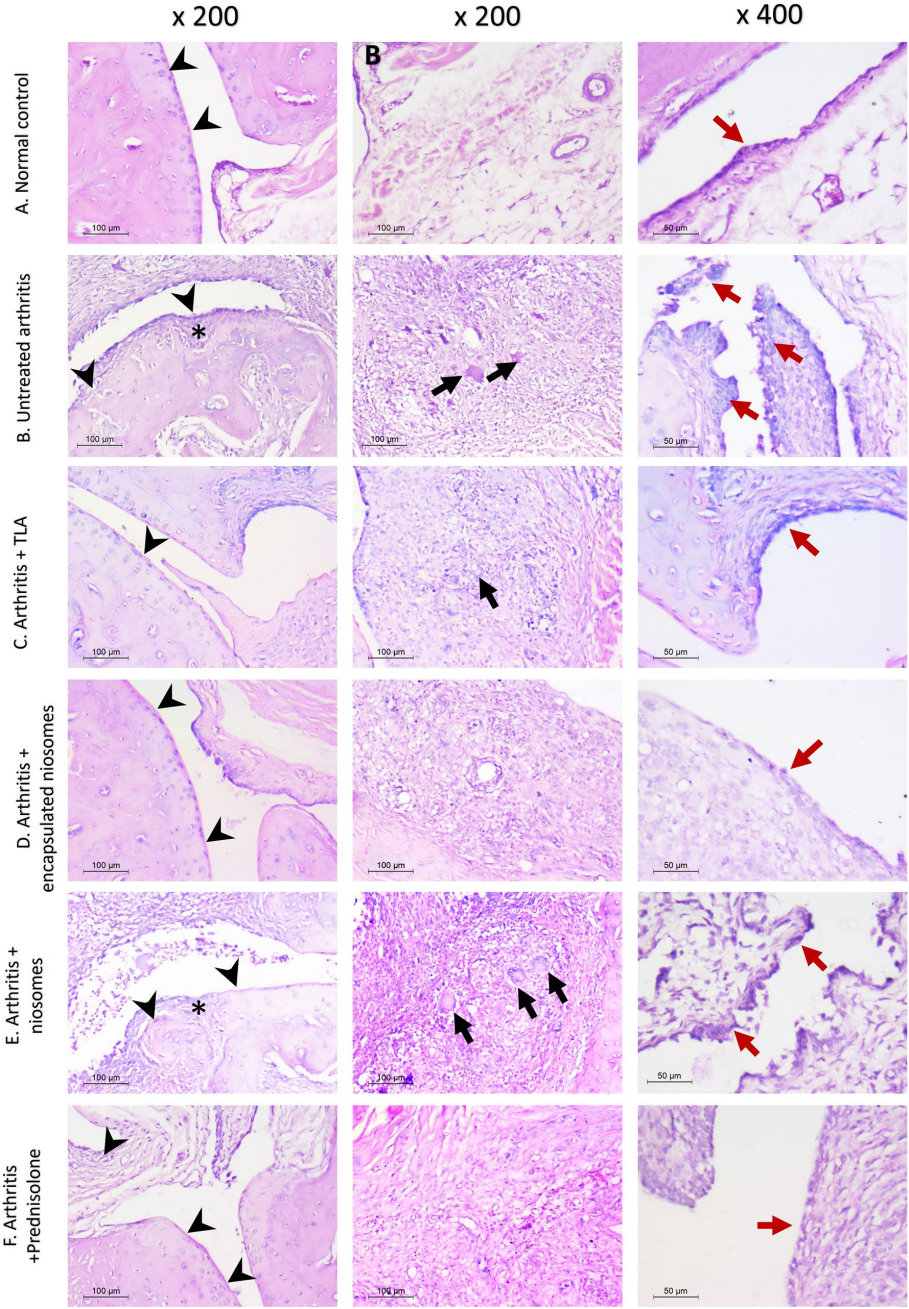
H&E-stained sections of hind paw of different studied groups. First column (× 200) demonstrates the cartilaginous surface (arrow heads) and the presence of bone erosions (*). Second column (× 200) represents the sub-synovial tissue infiltration by inflammatory cells with black arrows pointing at giant cells. third column (× 400) highlights the synovial lining (red arrow) to show the synovial hyperplasia. **A** Normal: showing intact cartilaginous surface (arrowheads) and loose sub-synovial tissue with no significant inflammation. High power shows thin synovial lining (red arrow) **B** Untreated arthritis: shows evident irregular cartilaginous surface (arrow heads) with underlying bone erosion (*). The sub-synovial tissue shows severe infiltration by lymphocytes and plasma cells with numerous giant cells (black arrow). High power view showing papillary synovial hyperplasia (red arrow) **C** TLA-treated arthritis: reveals restoration of cartilage with only minor irregularities (arrowhead). The sub-synovial tissue shows only moderate inflammation with occasional giant cells (black arrow). The synovial shows moderate hyperplasia (red arrow) **D** TLA-encapsulated niosomes-treated arthritis: reveals total restoration of cartilaginous surface (arrowheads). The sub-synovial tissue shows mild inflammation. The synovium is normal (red arrow), **F** Niosomes-treated arthritis: similar changes as untreated RA group with only minimal improvement. Irregular cartilaginous surface (arrowheads) and bone erosions (*) are clearly seen. The synovium is heavily inflamed with multiple giant cells (black arrows). The synovium is hyperplastic and papillary (red arrow). **G** Prednisolone-treated arthritis: reveals total restoration of cartilaginous surface (arrowheads). The sub-synovial tissue shows mild inflammation. The synovium is normal (red arrow) (color figure online)

The effect of both TLA and TLA-encapsulated niosomes-treated arthritis groups was comparable to prednisolone effect which showed good improvement of histopathologic changes with no statistically significant difference between them (*P* = 0.943 for both groups) (Table 1 and Fig. 5).

**Table 1:** Histopathologic findings in different studied groups

	Cartilage/ bone erosion Mean ± SD	Inflammation Mean ± SD	Pannus Mean ± SD	Hyperplasia Mean ± SD	Total score Mean ± SD	*P* value
Normal (*n* = 6)	0 ± 0	0 ± 0	0 ± 0	0 ± 0	0 ± 0	**< 0.001**
Untreated arthritis (*n* = 6)	3.17 ± 0.75	3.830 ± 0.41	3.830 ± 0.41	3.17 ± 0.75	14 ± 1.41	***P***_***0***_ = < 001*
Arthritis + TLA (*n* = 6)	0.50 ± 0.55	1.50 ± 0.55	0.67 ± 0.52	1.00 ± 0.89	3.67 ± 2.07	***P***_***0***_** = *0.051*** ***P***_***1***_ = 0.003*
Arthritis + TLA-encapsulated niosomes (*n* = 6)	0.33 ± 0.52	1.33 ± 0.52	0.83 ± 0.75	1.17 ± 0.75	3.67 ± 2.07	***P***_***0***_** = *0.051*** ***P***_***1***_ = 0.003* ***P***_***2***_** = *1.000***
**Arthritis + niosomes** (n = 6)	1.67 ± 0.52	3.33 ± 0.52	2.67 ± 0.52	2.67 ± 0.52	10.33 ± 1.37	***P***_***0***_ = < 0.001* ***P***_***1***_** = *0.349*** ***P***_***3***_ = 0.042* ***P***_***4***_ = 0.042*
**TLA + prednisolone** (n = 6)	0.50 ± 0.55	1.50 ± 0.55	0.50 ± 0.55	1.33 ± 0.52	3.83 ± 1.47	***P***_***0***_ = 0.042* ***P***_***1***_ = 0.004* ***P***_***5***_** = *0.934*** ***P***_***6***_** = *0.409*** ***P***_***7***_** = *0.51***

p_0_: p value for comparing between **normal control** and each other group, p_1_: p value for comparing between **untreated arthritis** and each other group, p_2_: p value for comparing between **arthritis + TLA** and **arthritis + TLA-encapsulated niosomes,** p_3_: p value for comparing between **RA + TLA** and **RA + niosomes,** p_4_: p value for comparing between **arthritis + TLA-encapsulated niosomes** and **arthritis + niosomes,** p_5_: p value for comparing between **arthritis + TLA** and **arthritis + prednisolone,** p_6_: p value for comparing between **arthritis + TLA-encapsulated niosomes** and **arthritis + prednisolone,** p_7_: p value for comparing between **arthritis + niosomes** and **arthritis + prednisolone,** *Statistically significant at p ≤ 0.05. *TLA Toxoplasma* lysate antigen

#### Immunohistochemistry

**Fig. 6:**
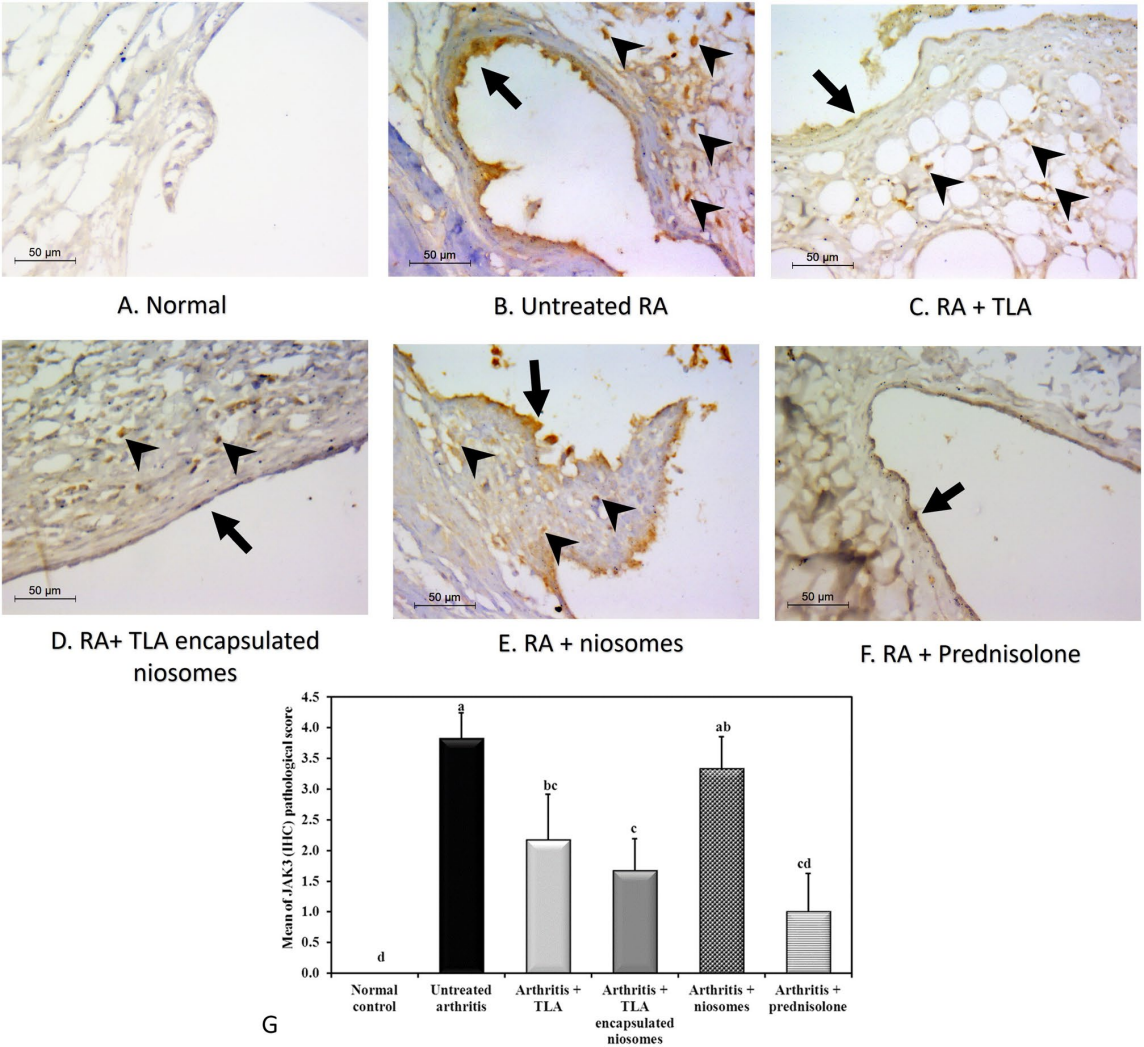
Assessment of JAK3 expression by immunohistochemistry in studied joints (× 400, IHC). Arrows points at synovial lining and arrowheads highlights expression in the inflammatory cells. **A** Normal: shows negative immunostaining of JAK3. **B** Untreated arthritis: reveals strong cytoplasmic positivity in synovial cells (arrow) and diffuse positivity in inflammatory cells (arrowheads). **C** Arthritis + TLA: decreased expression of JAK3 in both synovial lining (arrow) and inflammatory cells (arrowheads). **D** Arthritis + TLA-encapsulated niosomes: the synovial lining shows negative immunostaining and focal staining of inflammatory cells (arrowheads). **E** Arthritis + niosomes: strong diffuse immunostaining is seen in synovial lining (arrow) and slight decrease of immunostaining in inflammatory cells (arrowheads). **F** Prednisolone treated arthritis: focal expression of JAK3 in synovial lining (arrow) and no inflammatory cells are seen in the field. **G** Bar chart highlighting the different scores of JAK3 immunostaining in different studied groups. Means with any common letter from “a” to “d” are not significantly different, i.e., means with totally different letters “a” to “d” are significantly different

Normal joints did not express anti- JAK3 by immunohistochemistry. Meanwhile, evident expression was seen in untreated RA which was seen in the form of strong positivity in synovial cells (more than 75%, score 4) and weaker however diffuse positivity in inflammatory cells in sub-synovial tissue. Scoring was done in × 400 field. At least three power fields were assessed in each joint. Then a mean final score was given. This was statistically significant in comparison to normal group (*p* =  < 0.001). TLA treated arthritis group changed the anti-JAK3 mean score to be 2 while treatment with TLA-encapsulated niosomes in arthritis group changed the mean score to 1. Both scores were statistically significant in comparison to arthritis untreated group (*P* = 0.043 and 0.008, respectively). However, no significant change was seen between both groups (*p* = 0.529). The prednisolone-treated arthritis group which showed only focal positivity of anti-JAK3 expression (score 1–2) was slightly better than TLA and TLA-encapsulated niosomes groups; however, no statistical significance was seen with them (*P* = 0.146, 0.409, respectively). The niosomes-treated arthritis group slightly decreased the score 3–4 in most of rats; however, this was statistically insignificant in comparison to untreated arthritis (*P* = 0.557). (Fig. 6).

#### Toxicity study

Histopathological examination for different organs including liver, spleen and kidneys was done in treated groups for exclusion of any hazardous consequences of the used regimens. All examined material revealed normal architecture and histology of the studied organs.

## Discussion

Inflammatory arthritis in RA is a known debilitating condition caused by immune system disorder (Institute and of Arthritis and Musculoskeletal and Skin Diseases (NIH) 2014). The disease is treated with medications that suppress the immune system leading to remission of the disease, but those medications cause many side effects (Singh et al. 2011; Stuart 2020). This study was a trial to deal with the disordered immune system through immune modulation by introducing TLA and its incorporation with a nano-carrier (niosomes) to study their effects on disease activity in comparison to prednisolone.

The arthritis model was successfully induced and was evident in the hind paws of rats which received no treatment and rats were debilitated and there was a significant weight loss in this group. Male rats were preferred to be involved in the study to avoid influences of hormonal changes of female rats on inflammatory cytokines (Nunomura 1990). TLA parasitic antigen was introduced instead of *Toxoplasma gondii* to avoid giving the whole infection.

Results of this study showed evident anti-arthritic effect of TLA which corresponds the result of El-Maleky et al. study that the TLA by itself can produce immune responses when given as a vaccination (EL-Malky 2014) and the anti-arthritic effect of TLA agrees with studies which showed that effect for the total *Toxoplasma* infection (Washino et al. 2012) and gamma radiation-attenuated *Toxoplasma* infection on arthritis animal model (Hafez et al. 2020), the same effect was present in the current study on arthritis and on IL 17 decreased expression. While IL-10 which is an anti-inflammatory cytokine is upregulated with *Toxoplasma* infection, as discovered in many studies, to suppress host immunity for infection survival (Jeong et al. 2016; Neyer et al. 1997; Khan et al. 1995), the same effect appeared on arthritis model (Washino et al. 2012; Hafez et al. 2020). TLA showed its anti-inflammatory properties when tested in allergic inflammation of mice lungs (Neyer et al. 1997) which agrees with this study findings. It should be put in mind that although *Toxoplasma* infection has immune-suppressive effects through different mechanisms, it also enhances immune response by which host immune system tries to fight infection (Sasai and Yamamoto 2019) including increased IL-17 expression (Guiton et al. 2010; Kelly et al. 2005). Another contrary hypothesis to our findings is that *Toxoplasma* may be a trigger for the disease and this was attributed to high prevalence of *Toxoplasma* antibodies among RA patients where some studies showing this finding concluded that toxoplasmosis may be a risk factor for RA but this is still controversial (Hosseininejad et al. 2018) Some studies explained it in another way that this is a result of immune-suppressive medications the patient is receiving which increases liability to infections (El-Henawy et al. 2017). Another explanation is that this may be owing to the presence of false-positive antibodies for infections produced in various autoimmune diseases due immune disorders and cross reactivity (Hsieh et al. 2007; Weiss et al. 1995).

TLA -encapsulated niosomes has given better results than that of TLA alone; this could be explained by the fact that niosomes increase bioavailability of the carried substance (Moghassemi and Hadjizadeh 2014; Mujeeb et al. 2015). Both TLA and TLA-encapsulated niosomes anti-inflammatory results were comparable to those of prednisolone. TLA and TLA-encapsulated niosomes, especially the latter, may have given better results if were given a bigger chance instead of the short experiment duration which is not possible to be held on a longer period; as arthritis signs disappeared when the model was left for more time in many references (Nasuti et al. 2019), also encapsulation efficiency of more than 75% would improve TLA-encapsulated niosomes results.

For niosomes group, it was involved in the study design for comparison of its effects with that of TLA and TLA-encapsulated niosomes regarding immune modulation and anti-inflammatory actions, as this effect of niosomes was described in some studies that showed not only inhibitory but also stimulatory effects on immunity (Gogoi et al. 2018; Brewer and Alexander 1992). This study results showed that niosomes obviously lead to anti-inflammatory anti-arthritic effects but was much less than the effect of TLA alone or TLA-encapsulated niosomes.

Study of Chen et al. (Chen et al. 2018) result is agreeing with the study result of the inhibitory effect TLA and TLA-encapsulated niosomes on JAK3 where Chen et al. noticed that *Toxoplasma* excreted-secreted antigens (ESA) inhibited JAK3 (Kumar et al. 2021) to which the targeted inhibitory medication tofacitinib is applied for RA treatment, confirming the anti-arthritic anti-inflammatory role of TLA.

Application of TLA and TLA-encapsulated niosomes for the treatment of inflammatory arthritis, i.e., RA and other autoimmune diseases should be studied well before introduction in patients to avoid any side effects. If the process was successful, and the anti-inflammatory effects were obvious, this would change the future of immunomodulatory treatment of autoimmune diseases.

## Conclusion

Vaccination with TLA or TLA-encapsulated niosomes might be useful for immune modulation of arthritis model of RA.

## Recommendations

Still efficacy, safety and exclusion of hazards should be studied on a larger base to test for the possibility of their introduction for disease treatment and in other autoimmune diseases.

### Supplementary Information

Below is the link to the electronic supplementary material.

Supplementary file1 A: Successful induction of arthritis. B: Measurement of hind paw thickness using Vernier caliper. (TIF 2667 KB)

Supplementary file2 shows IL-10 and IL-17 expression in all groups and supp. Fig. 3: shows that of CRP. Means with any common letter from “a” to “d” are not significantly different, i.e., means with totally different letters “a” to “d” are significantly different. (JPG 110 KB)

Supplementary file3 (JPG 72 KB)

## Data Availability

Available on request.

## Abbreviations

RA: Rheumatoid arthritis
NSAIDs: Nonsteroidal anti-inflammatory drugs
DMARDs: Disease-modifying anti-rheumatic drugs
*T gondii*: *Toxoplasma gondii*
JAK3: Janus kinase 3
IL: Interleukin
ROP16: Rhoptry kinase 16
TLA: *toxoplasma* Lysate antigen
CFA: Complete Freund’s Adjuvant
PBS: Phosphate-buffered saline

## References

[CR1] Aggarwal S, Gurney AL (2002). IL-17: prototype member of an emerging cytokine family. J Leukoc Biol.

[CR2] Brewer JM, Alexander J (1992). The adjuvant activity of non-ionic surfactant vesicles (niosomes) on the BALB/c humoral response to bovine serum albumin. Immunology.

[CR3] Chen J, Huang C, Zhu D, Chen L, Wang J, Sun X, Hu L, Duan Y (2018). Excreted-secreted antigens of Toxoplasma gondii inhibit Foxp3 via IL-2Rγ/JAK3/Stats pathway. J Cell Biochem.

[CR4] Cremer MA, Townes AS, Kang AH (1990). Adjuvant-induced arthritis in rats evidence that autoimmunity to homologous collagens types i, II, IX and XI is not involved in the pathogenesis of arthritis. Clin Exp Immunol.

[CR5] Darwish SF, El-Bakly WM, Arafa HM, El-Demerdash E (2013). Targeting TNF-α and NF-κB activation by bee venom: role in suppressing adjuvant induced arthritis and methotrexate hepatotoxicity in rats. PLoS One.

[CR6] El Temsahy MM, El Kerdany ED, Eissa MM, Shalaby TI, Talaat IM, Mogahed NM (2016). The effect of chitosan nanospheres on the immunogenicity of toxoplasma lysate vaccine in mice. J Parasit Dis.

[CR7] El-Henawy AA, Hafez EAR, Nabih N, Shalaby NM, Mashaly M (2017). Anti-Toxoplasma antibodies in Egyptian rheumatoid arthritis patients. Rheumatol Int.

[CR8] El-Malky MA (2014). Vaccination with Toxoplasma lysate antigen and CpG oligodeoxynucleotides: comparison of immune responses in intranasal versus intramuscular administrations. Parasitol Res.

[CR9] Engwerda CR, Ng SS, Bunn PT (2014). The Regulation of CD4(+) T Cell Responses during Protozoan Infections. Front Immunol.

[CR10] Eskdale J, Kube D, Tesch H, Gallagher G (1997). Mapping of the human IL10 gene and further characterization of the 5' flanking sequence. Immunogenetics.

[CR11] GDB., PVL. (2020) Recent advances of non-ionic surfactant-based nano-vesicles (niosomes and proniosomes): a brief review of these in enhancing transdermal delivery of drug. Futur J Pharm Sci 6, 100 10.1186/s43094-020-00117-y.

[CR12] Gogoi H, Mani R, Bhatnagar R (2018). A niosome formulation modulates the Th1/Th2 bias immune response in mice and also provides protection against anthrax spore challenge. Int J Nanomedicine.

[CR13] Guiton R, Vasseur V, Charron S, Arias MT, Van Langendonck N, Buzoni-Gatel D, Ryffel B, Dimier-Poisson I (2010). Interleukin 17 receptor signaling is deleterious during Toxoplasma gondii infection in susceptible BL6 mice. J Infect Dis.

[CR14] Hafez EN, Moawed FSM, Abdel-Hamid GR, Eldin ES (2020). Immunomodulatory activity of gamma radiation-attenuated Toxoplasma gondii in adjuvant arthritic mice. J. Photochem Photobiol B.

[CR15] Hasseldam H, Hansen CS, Johansen FF (2013). Immunomodulatory effects of helminths and protozoa in multiple sclerosis and experimental autoimmune encephalomyelitis. Parasite Immunol.

[CR16] Hosseininejad Z, Sharif M, Sarvi S, Amouei A, Hosseini SA, Nayeri Chegeni T, Anvari D, Saberi R, Gohardehi S, Mizani A, Sadeghi M, Daryani A (2018). Toxoplasmosis seroprevalence in rheumatoid arthritis patients: A systematic review and meta-analysis. PLoS Negl Trop Dis.

[CR17] Hsieh YF, Liu HW, Hsu TC, Wei JC, Shih CM, Krause PJ, Tsay GJ (2007). Serum reactivity against Borrelia burgdorferi OspA in patients with rheumatoid arthritis. Clin Vaccine Immunol.

[CR18] National Institute for Health and Care Excellence (NICE), (2020) Rheumatoid arthritis in adults: management: Recommendations: Guidance and guidelines, NICE, UK,.

[CR19] National Institute of Arthritis and Musculoskeletal and Skin Diseases (NIH), (2014) Handout on Health: Rheumatoid Arthritis, NIH, Bethesda, Maryland.

[CR20] Jackson JA, Friberg IM, Little S, Bradley JE (2009). Review series on helminths, immune modulation and the hygiene hypothesis: immunity against helminths and immunological phenomena in modern human populations: coevolutionary legacies?. Immunology.

[CR21] Jankovic D, Kullberg MC, Feng CG, Goldszmid RS, Collazo CM, Wilson M, Wynn TA, Kamanaka M, Flavell RA, Sher A (2007). Conventional T-bet(+)Foxp3(-) Th1 cells are the major source of host-protective regulatory IL-10 during intracellular protozoan infection. J Exp Med.

[CR22] Jensen KD, Hu K, Whitmarsh RJ, Hassan MA, Julien L, Lu D, Chen L, Hunter CA, Saeij JP (2013). Toxoplasma gondii rhoptry 16 kinase promotes host resistance to oral infection and intestinal inflammation only in the context of the dense granule protein GRA15. Infect Immun.

[CR23] Jeong YI, Hong SH, Cho SH, Park MY, Lee SE (2016). Induction of IL-10-producing regulatory B cells following Toxoplasma gondii infection is important to the cyst formation. Biochem Biophys Rep.

[CR24] Kelly MN, Kolls JK, Happel K, Schwartzman JD, Schwarzenberger P, Combe C, Moretto M, Khan IA (2005). Interleukin-17/interleukin-17 receptor-mediated signaling is important for generation of an optimal polymorphonuclear response against Toxoplasma gondii infection. Infect Immun.

[CR25] Khan IA, Matsuura T, Kasper LH (1995). IL-10 mediates immunosuppression following primary infection with Toxoplasma gondii in mice. Parasite Immunol.

[CR26] Kumar N, Kuang L, Villa R, Kumar P, Mishra J (2021). Mucosal epithelial Jak kinases in health and diseases. Mediators Inflamm.

[CR27] Kuncha M, Naidu VG, Sahu BD, Gadepalli SG, Sistla R (2014). Curcumin potentiates the anti-arthritic effect of prednisolone in Freund's complete adjuvant-induced arthritic rats, J. Pharm Pharmacol.

[CR28] Lima TS, Lodoen MB (2019). Mechanisms of human innate immune evasion by Toxoplasma gondii. Front Cell Infect Microbiol.

[CR29] Lüder CG, Campos-Salinas J, Gonzalez-Rey E, van Zandbergen G (2010). Impact of protozoan cell death on parasite-host interactions and pathogenesis. Parasit Vectors.

[CR30] Maizels RM (2009). Parasite immunomodulation and polymorphisms of the immune system. J Biol.

[CR31] Majithia V, Geraci SA (2007). Rheumatoid arthritis: diagnosis and management. Am J Med.

[CR32] Miossec P, Kolls JK (2012). Targeting IL-17 and TH17 cells in chronic inflammation. Nat Rev Drug Discov.

[CR33] Moghassemi S, Hadjizadeh A (2014). Nano-niosomes as nanoscale drug delivery systems: an illustrated review. J Control Release.

[CR34] Mujeeb S, Krishna Sailaja A (2015). Niosomes: A vesicular system for drug targeting. J. Pharm. Biol Sci.

[CR35] Nasuti C, Fedeli D, Bordoni L, Piangerelli M, Servili M, Selvaggini R, Gabbianelli R (2019). Anti-inflammatory, anti-arthritic and Anti-nociceptive activities of Nigella sativa Oil in a rat model of arthritis. Antioxidants (basel).

[CR36] Neyer LE, Grunig G, Fort M, Remington JS, Rennick D, Hunter CA (1997). Role of interleukin-10 in regulation of *T*-cell-dependent and *T*-cell-independent mechanisms of resistance to Toxoplasma gondii. Infect Immun.

[CR37] Nunomura W (1990). C-reactive protein in rat: in development, pregnancy and effect of sex hormones. Comp Biochem Physiol A Comp Physiol.

[CR38] Osada Y, Shimizu S, Kumagai T, Yamada S, Kanazawa T (2009). Schistosoma mansoni infection reduces severity of collagen-induced arthritis via down-regulation of pro-inflammatory mediators. Int J Parasitol.

[CR39] Pardakhty A, Shakibaie M, Daneshvar H, Khamesipour A, Mohammadi-Khorsand T, Forootanfar H (2012). Preparation and evaluation of niosomes containing autoclaved Leishmania major: a preliminary study. J Microencapsul.

[CR40] Sasai M, Yamamoto M (2019). Innate, adaptive, and cell-autonomous immunity against Toxoplasma gondii infection. Exp Mol Med.

[CR41] Shirani K, Iranshahi M, Askari VR, Gholizadeh Z, Zadeh AA, Zeinali M, Hassani FV, Taherzadeh Z (2021). Comparative evaluation of the protective effects of oral administration of auraptene and umbelliprenin against CFA-induced chronic inflammation with polyarthritis in rats. Biomed. Pharmacother..

[CR42] Silva MD, Savinainen A, Kapadia R, Ruan J, Siebert E, Avitahl N, Mosher R, Anderson K, Jaffee B, Schopf L, Chandra S (2004). Quantitative analysis of micro-CT imaging and histopathological signatures of experimental arthritis in rats. Mol Imaging.

[CR43] Singh JA, Saag KG, Bridges SL, Akl EA, Bannuru RR, Sullivan MC, Vaysbrot E, McNaughton C, Osani M, Shmerling RH, Curtis JR, Furst DE, Parks D, Kavanaugh A, O'Dell J, King C, Leong A, Matteson EL, Schousboe JT, Drevlow B, Ginsberg S, Grober J, St Clair EW, Tindall E, Miller AS, McAlindon T (2015). American college of rheumatology guideline for the treatment of rheumatoid arthritis. Arthritis Rheumatol.

[CR44] Singh JA, Wells GA, Christensen R, Tanjong Ghogomu E, Maxwell L, Macdonald JK, Filippini G, Skoetz N, Francis D, Lopes LC, Guyatt GH, Schmitt J, La Mantia L, Weberschock T, Roos JF, Siebert H, Hershan S, Lunn MP, Tugwell P, Buchbinder R (2011). Adverse effects of biologics: a network meta-analysis and Cochrane overview. Cochrane Database Syst Rev.

[CR45] Smallwood TB, Giacomin PR, Loukas A, Mulvenna JP, Clark RJ, Miles JJ (2017). Helminth immunomodulation in autoimmune disease. Front Immunol.

[CR46] A. Stuart, Rheumatoid Arthritis Drug Guide. https://www.webmd.com/rheumatoid-arthritis/rheumatoid-arthritis-medications, 2020 (Accessed 13 Oct 2022)

[CR47] Walker JG, Ahern MJ, Coleman M, Weedon H, Papangelis V, Beroukas D, Roberts-Thomson PJ, Smith MD (2006). Expression of Jak3, STAT1, STAT4, and STAT6 in inflammatory arthritis: unique Jak3 and STAT4 expression in dendritic cells in seropositive rheumatoid arthritis. Ann Rheum Dis.

[CR48] Washino T, Moroda M, Iwakura Y, Aosai F (2012). Toxoplasma gondii infection inhibits Th17-mediated spontaneous development of arthritis in interleukin-1 receptor antagonist-deficient mice. Infect Immun.

[CR49] Weiss NL, Sadock VA, Sigal LH, Phillips M, Merryman PF, Abramson SB (1995). False positive seroreactivity to Borrelia burgdorferi in systemic lupus erythematosus: the value of immunoblot analysis. Lupus.

[CR50] Wilson EH, Wille-Reece U, Dzierszinski F, Hunter CA (2005). A critical role for IL-10 in limiting inflammation during toxoplasmic encephalitis. J Neuroimmunol.

