# Supplementary figures and images for: Vaccination with Toxoplasma lysate antigen or its encapsulated niosomes form immunomodulates adjuvant-induced arthritis through JAK3 downregulation
Source: Inflammopharmacology. 2023 Jun 30;31(6):3101–14. doi: 10.1007/s10787-023-01267-0 (PMC10692027; doi:10.1007/s10787-023-01267-0)

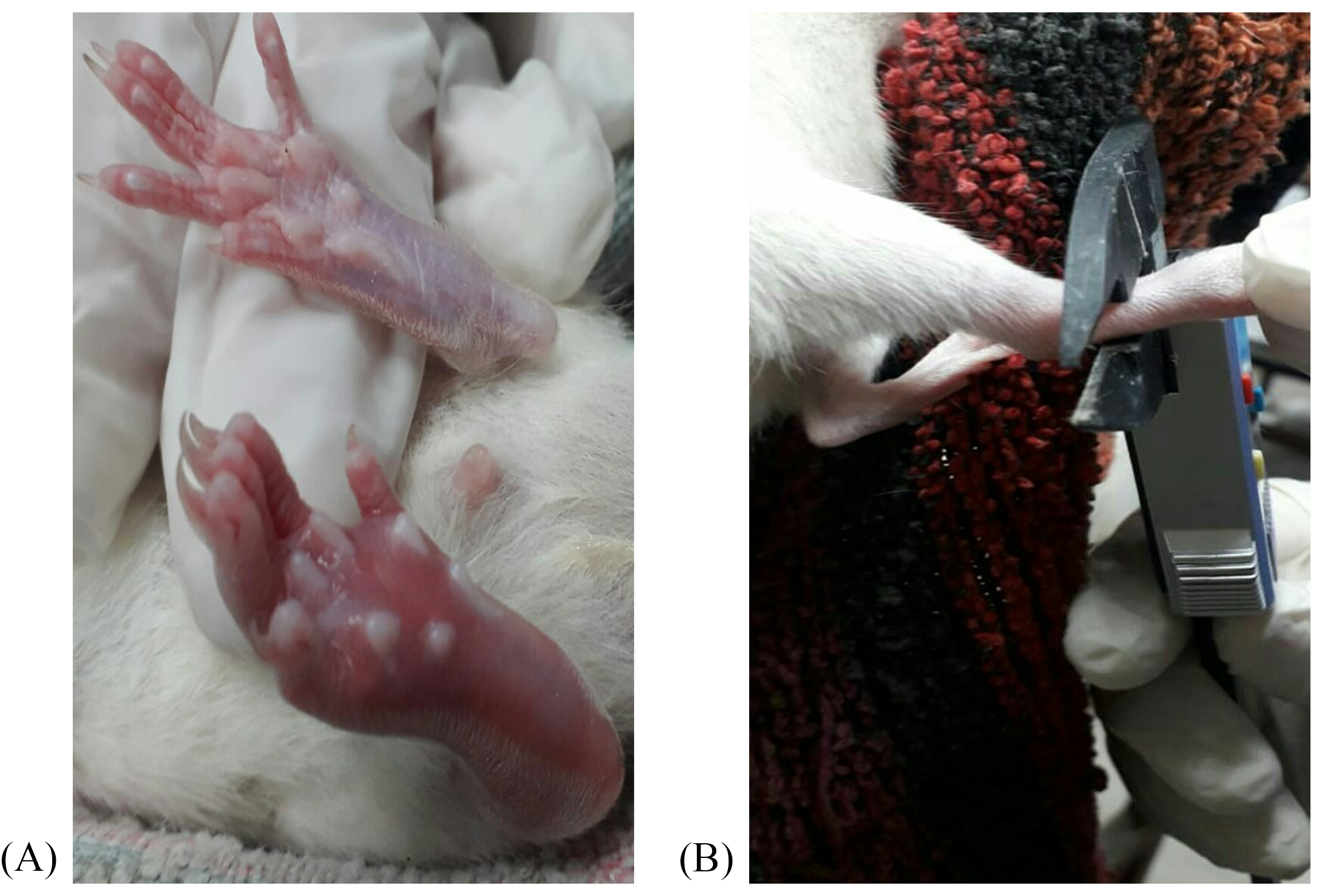

Supplement: Supplementary file 1 — Supplementary file1 A: Successful induction of arthritis. B: Measurement of hind paw thickness using Vernier caliper. (TIF 2667 KB) [file 10787_2023_1267_MOESM1_ESM.tif]

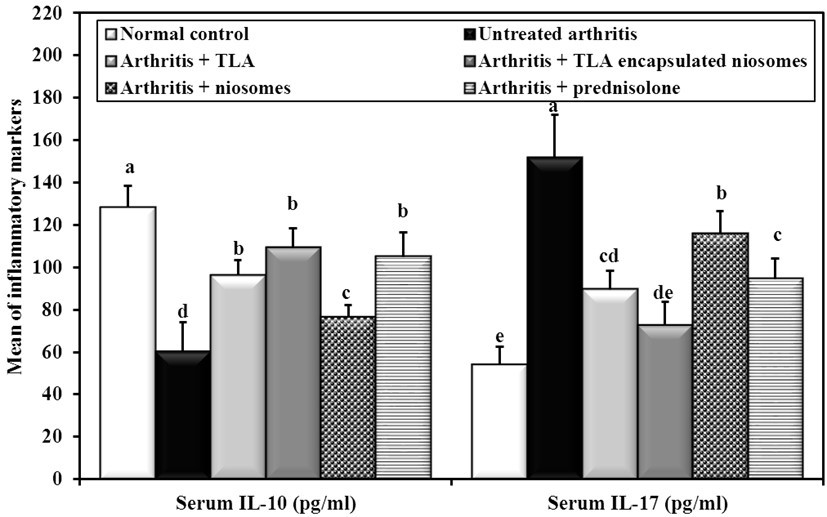

Supplement: Supplementary file 2 — Supplementary file2 shows IL-10 and IL-17 expression in all groups and supp. Fig. 3: shows that of CRP. Means with any common letter from “a” to “d” are not significantly different, i.e., means with totally different letters “a” to “d” are significantly different. (JPG 110 KB) [file 10787_2023_1267_MOESM2_ESM.jpg]

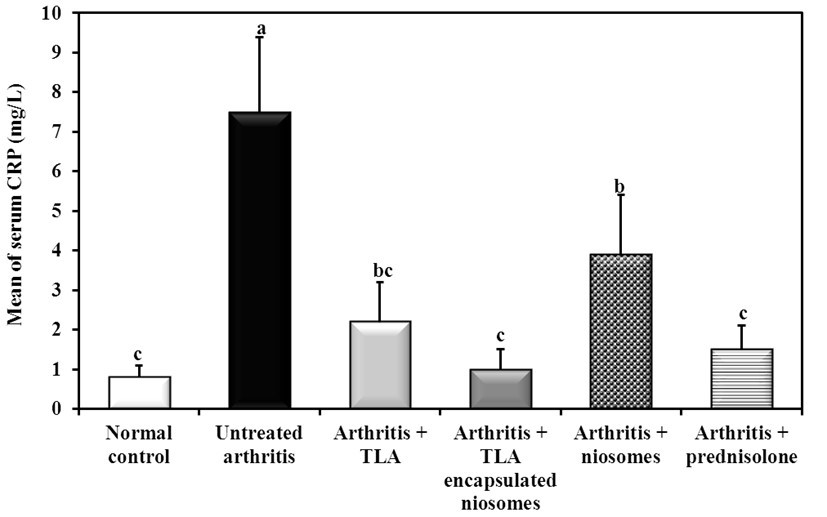

Supplement: Supplementary file 3 — Supplementary file3 (JPG 72 KB) [file 10787_2023_1267_MOESM3_ESM.jpg]
